# Interaction of the primordial germ cell-specific protein C2EIP with PTCH2 directs differentiation of embryonic stem cells via HH signaling activation

**DOI:** 10.1038/s41419-018-0557-2

**Published:** 2018-04-27

**Authors:** Qisheng Zuo, Kai Jin, Jiuzhou Song, Yani Zhang, Guohong Chen, Bichun Li

**Affiliations:** 1 Key Laboratory of Animal Breeding Reproduction and Molecular Design for Jiangsu Province, College of Animal Science and Technology, Yangzhou University, Yangzhou, Jiangsu 225009 P.R. China; 2 Animal & Avian Sciences, University of Maryland, Baltimore, MD 20741 USA

## Abstract

Although many marker genes for germ cell differentiation have been identified, genes that specifically regulate primordial germ cell (PGC) generation are more difficult to determine. In the current study, we confirmed that *C2EIP* is a PGC marker gene that regulates differentiation by influencing the expression of pluripotency-associated genes such as *Oct4* and *Sox2*. Knockout of *C2EIP* during embryonic development reduced PGC generation efficiency 1.5-fold, whereas *C2EIP* overexpression nearly doubled the generation efficiency both in vitro and in vivo. *C2EIP* encodes a cytoplasmic protein that interacted with PTCH2 at the intracellular membrane, promoted PTCH2 ubiquitination, activated the Hedgehog (HH) signaling pathway via competitive inhibition of the GPCR-like protein SMO, and positively regulated PGC generation. Activation and expression of *C2EIP* are regulated by the transcription factor STAT1, histone acetylation, and promoter methylation. Our data suggest that *C2EIP* is a novel, specific indicator of PGC generation whose gene product regulates embryonic stem cell differentiation by activating the HH signaling pathway via PTCH2 modification.

## Introduction

Primordial germ cells (PGC) can be used for therapeutic purposes and genetic modification as an alternative to embryonic stem cells^1,2^. Unfortunately, harvesting plentiful PGC in vitro is often problematic because of the limited understanding of their ontogenetic mechanism, which greatly confines their clinical application^3^. Multiple gene products are specifically involved in PGC differentiation^4^. For example, hypoxia induces PGC generation by maintaining HIF1 expression and down-regulating *Oct4* expression^5,6^, whereas Blimp1 and Prdm14 regulate PGC generation in mice by epigenetically modifying and re-programming them to differentiate into spermatogonial stem cells (SSC)^7–10^. Moreover, RNAi-mediated knockout of LIN28 downregulates expression of multiple germ cell surface markers during STELLA^+^ cell differentiation, including key PGC development genes such as *Blimp1*, *Prdm14*, and endogenous *STELLA*^11,12^.Although these genes can mark PGC and be used to track their migration and colonization, not all of them are uniquely expressed in PGC and their regulatory mechanisms are not conspicuous.

In the present study, we identified a novel PGC-specific marker gene, *C2EIP*^13,14^, and characterized its molecular function in detail. We previously reported that *C2EIP* was not noticeably expressed in embryonic stem cells (ESC), SSC, or chick embryo fibroblasts but are specifically expressed in PGC. Therefore, we speculated that *C2EIP* is likely a novel key genetic marker for PGC generation and differentiation. This hypothesis is supported by earlier studies showing that CRISPR/Cas9-mediated knockout of *C2EIP* in chick embryos significantly limited their development^13^ and reduced PGC generation efficiency, and *C2EIP* overexpression promoted PGC generation. The GST pull-down assay showed that C2EIP interacted with PTCH2, an important membrane protein of the Hedgehog (HH) signaling pathway, which can upregulate germ cell differentiation and PGC generation^15^. However, whether *C2EIP* function is associated with HH signal activation remains unknown. Thus, we investigated the ubiquitination and dephosphorylation status of PTCH2 in response to different expression levels of C2EIP. We found that *C2EIP* overexpression induced ubiquitinationand dephosphorylation of PTCH2 and upregulated PGC generation by releasing SMO protein and activating the HH signaling pathway. We also determined that *C2EIP* expression was regulated by transcription factor STAT1 as well asmethylation and acetylation patterns. In summary, we conclude that *C2EIP* is a novel, PGC-specific marker gene that regulates PGCgeneration in vivo and in vitro.

## Results

### *C2EIP* encodes a cytoplasmic protein that is differentially expressed in PGC

**Fig. 1:**
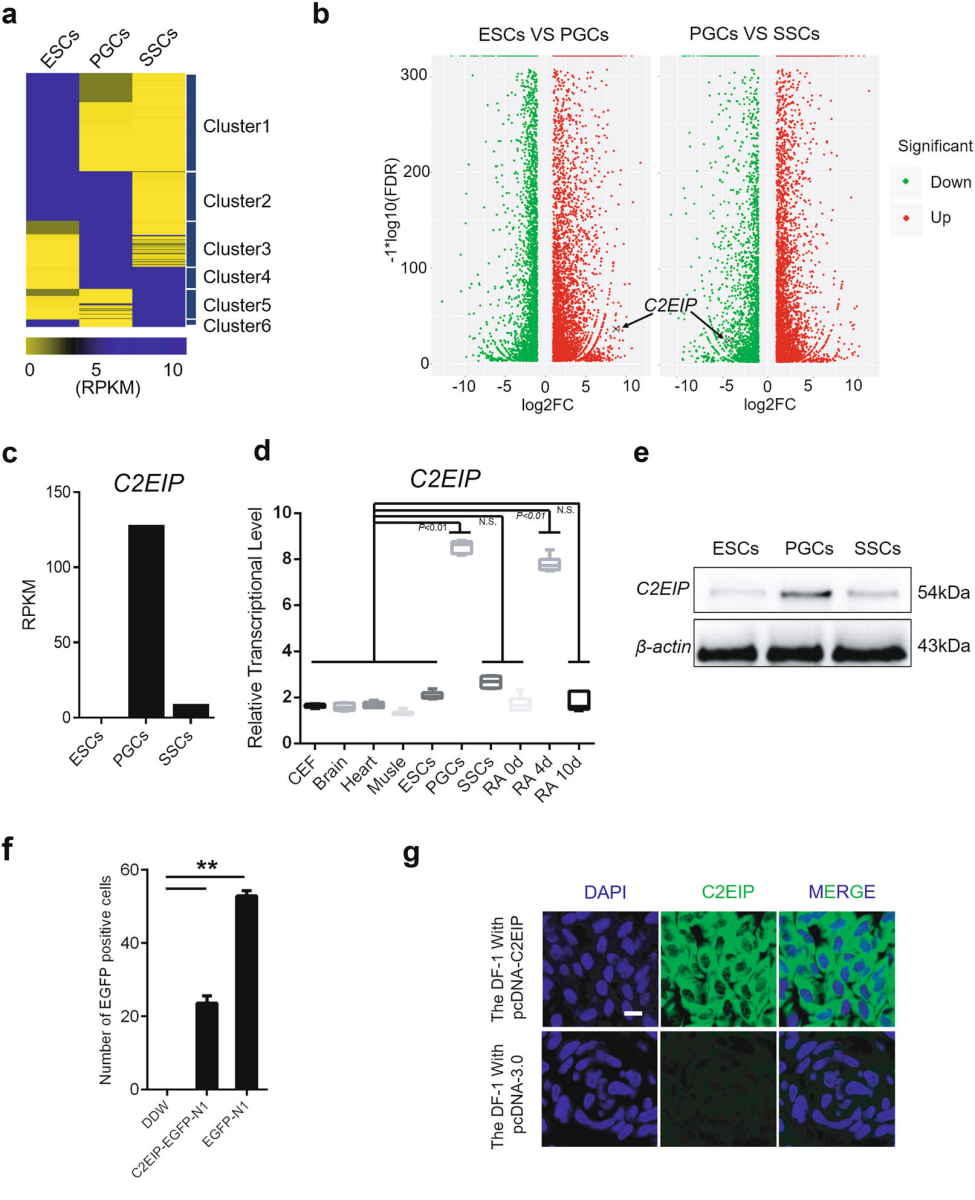
*C2EIP* encodes a cytoplasmic protein that is differentially expressed in PGC. **a** Transcriptome analysis of ESC, PGC, and SSC. The differential expression of genes in these cells can be divided into six clusters.Cluster1 represents genes with high expression in ESC but low expression in PGC and SSC; Cluster2 represents genes with high expression in ESC and PGC but low expression in SSC; Cluster3 represents genes with high expression in PGC but low expression in ESC and SSC; Cluster4 represents genes with high expression in PGC and SSC but low expression in ESC; Cluster5 represents genes with high expression in SSC but low expression in ESC and PGC; Cluster6 represents genes with high expression in ESC and SSC but low expression in PGC. **b** The distribution of differentially expressed genes in ESC vs. PGC and PGC vs. SSC indicate C2EIP is highly expressed in PGC. Red represents upregulated genes, green represents down-regulated genes. **c** The RPKM value of *C2EIP*, representing relative expression, in ESC, PGC, and SSC. **d** qRT-PCR analysis of C2EIP expression in indicated tissues, ESC, PGC, SSC, and RA-induced cells. **e** Western blot analysis of C2EIP protein levelsESC, PGC, and SSC.β-actin was used as an internal reference. **f** Confirmation of C2EIP-EGFP fusion protein expression. Cells were transfected with EGFP-N1 vector as a positive control, or mock-transfected with double distilled water (DDW) as a negative control. **g** Indirect immunofluorescence showing cytoplasmic localization of C2EIP. Cells transfected with pcDNA3.0 vector were used as a negative control, Scale bar:20μm

PGC are the ancestral cells of male germ cells^16,17^, and our research group has previously studied their migration, colonization, and differentiation^18^. Bioinformatic analyses revealed that *C2EIP* is located on the second chromosome and contains a 648-bp coding sequence (CDS) that encodes 215 amino acids with a relative molecular weight of 24.26 kD. C2EIP is a hydrophilic, non-secretory protein situated mainly in the cytoplasm that does not include a signal peptide sequence or special structural domain. Previous RNA-seq results showed that expression of *C2EIP* (XM-001233327.1, Chr2) is PGC-specific (Figs. 1a, b) (Supplementary Figure 1a, b)^13^. High-throughput sequencing results showed that the expression of *C2EIP* in PGCwas significantly higher than that in ESC and SSC, in which there was little to no *C2EIP* expression (Fig. 1c). Western blotting and qRT-PCR showed that expression of *C2EIP* in PGC was higher than in ESC and SSC (Figs. 1d, e), a finding supported by in vitro retinoic acid (RA) induction experiments^19,20^ that determined *C2EIP* expression was upregulated 5.5-fold compared to non-RA-induced cells (Fig. 1d). The stop codon of *C2EIP* was mutated, and a C2EIP-EGFP-N1 fusion expression vector was constructed to confirm that *C2EIP* successfully encoded protein in DF-1 cells (Fig. 1f) (Supplementary Figure 1c). To further investigate the subcellular localization of C2EIP proteins, we prepared a polyclonal antibody. We constructed a pcDNA3.0-C2EIP vector and injected it into mice intraperitoneally. The antibody titer was 1:10 (Supplementary Figure 1d). pcDNA3.0-C2EIP vector was alsotransfected intowell-cultured DF-1 cells and indirect immunofluorescence experiments were performed usingthe polyclonal antibody. The results showed that C2EIP was indeed expressed in the cytoplasm (Fig. 1g), and similar results were also found in PGC (Supplementary Figure 1e). Our results suggested that C2EIP was a PGC-specific gene that could encode protein.

### *C2EIP* is highly expressed in pgc via promoter and histone modification

**Fig. 2:**
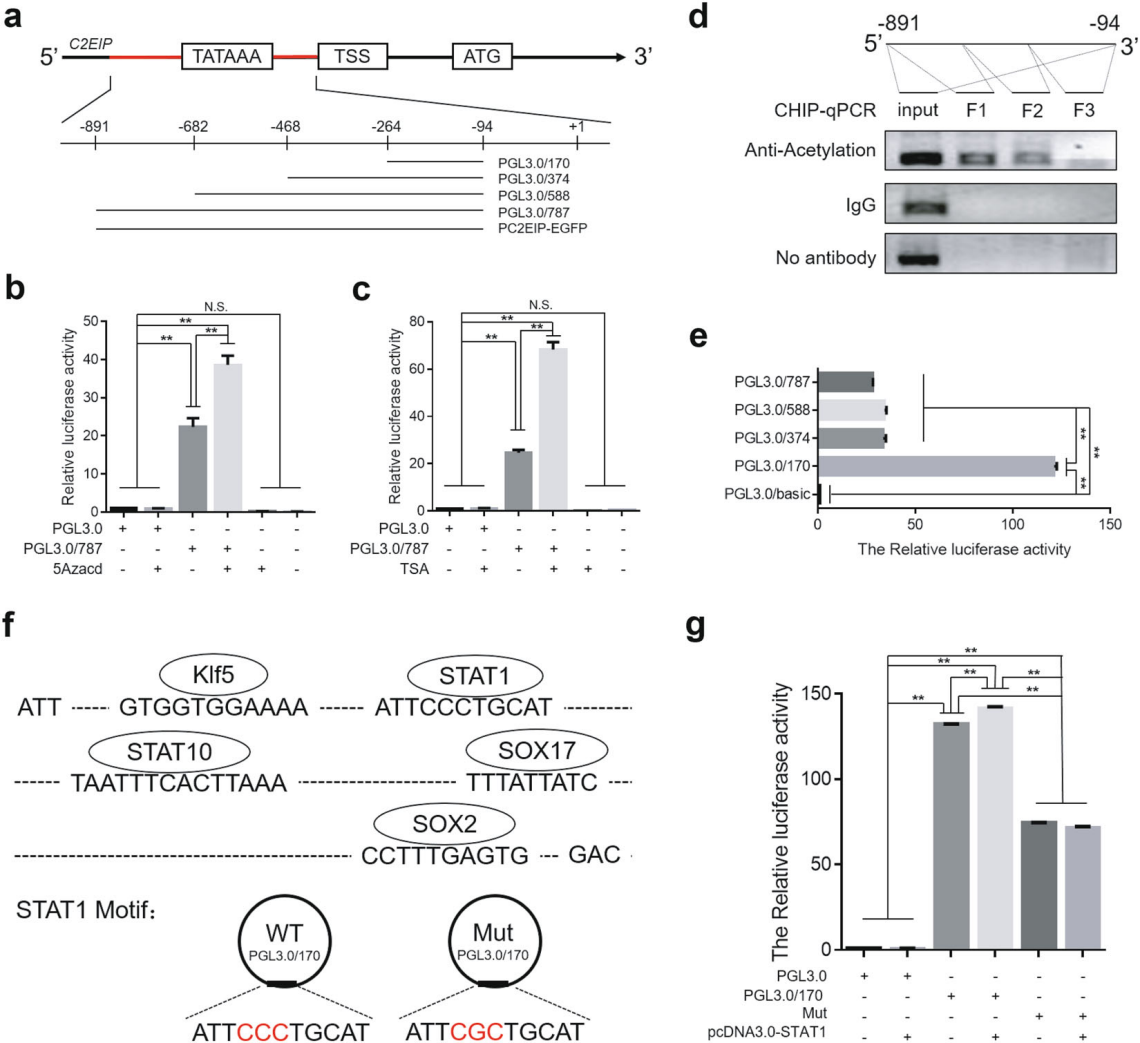
High *C2EIP* expression in PGC is mediated by promoter histone modification. **a** Schematic diagram of *C2EIP* promoter fragment cloning and vector construction. The double luciferase reporter system was used to examine the effects of 5-Zacd (**b**) and TSA (**c**) on *C2EIP* promoter activity. **d** CHIP-qPCR was used to detect histone acetylation in the *C2EIP* promoter region. **e** Luciferase activity in DF-1 co-transfected with PGL3.0/787, PGL3.0/588, PGL3.0/347,or PGL3.0/170. DF-1 cells transfected with PGL3.0/basic was a negative control. **f** Top:the location of STAT1, STAT10, Sox17, Sox2, and Klf5 in the core region of the C2EIP promoter; Bottom:diagram of STAT1 transcription factor binding site mutation. **g** The double luciferase reporter system was used to assess *C2EIP* promoter activity when the STAT1 transcription factor binding site was mutated (Mut) or STAT1 was overexpressed from the pcDNA3.0-STAT1 plasmid

Because we observed *C2EIP* more highly expressed in PGC than in ESC or SSC, we speculated that the activity of the *C2EIP* promoter changes during PGC generation, which leads to differential gene expression in PGC compared to ESC and SSC. Therefore, we identified the promoter region of the *C2EIP* gene based on the original promoter TATAbox and CAATbox and cloned the *C2EIP* promoter fragment to replace the *pEGFP-N1* promoter in a pC2EIP-EGFP recombinant vector. The constructed pC2EIP-EGFP vector (Fig. 2a) can express green fluorescent protein in DF-1 cells (Supplementary Figure 2a), confirming the *C2EIP* promoter activity.

Studies have shown that epigenetic factors (DNA methylation, histone acetylation, and transcription factors) affect promoter activity. We predicted, by bioinformatics analysis, that the cloned *C2EIP* promoter fragment does not contain CpG islands but does contain a small number of CG loci, suggesting that the *C2EIP* gene promoter activity is not likely to be affected by DNA methylation. To verify this hypothesis, we linked the *C2EIP* promoter fragment into the pGL3-Basic vector to construct the pGL3-787 recombinant vector, which we transfected into DF-1 cells that we treated with or without 10 μmol/L DNA methylation inhibitor (5-Zacd). The cells were collected for the dual luciferase reporter assay, which showed that the inhibition of methylation resulted in significant upregulation (37.24 ± 3.24) of *C2EIP* promoter activity compared to it without 5-Zacd treatment(23.41 ± 2.13), indicating that although the *C2EIP* promoter region does not contain a CpG island, DNA methylation can be mediated by the CG loci and affects promoter activity(Fig. 2b). *C2EIP* gene expression was detected in cultured PGC after treatment with 5-Zacd, which significant increased *C2EIP* expression, confirming the luciferase assayresults (Supplementary Figure 2b).While screening for other epigenetic factors affecting the activity of the *C2EIP* promoter, it was found that treatment of pGL3-787-transfected DF-1 cells with10 μmol/L of the histone deacetylase inhibitor TSA significantly upregulated*C2EIP* promoter activity (68.47 ± 3.11)compared to it without TSA treatment (22.35 ± 1.48) (Fig. 2c), indicating that histone acetylation can increase *C2EIP* promoter activity. CHIP-qPCR results also show that histone acetylation was enriched in the *C2EIP* promoter region (Fig. 2d).

The −264~−94 bp region was identified as the core region of the *C2EIP* promoter (Fig. 2e) based on the luciferase activity of truncated fragments (Fig. 2a) (Supplementary Figure 2c), and this region includesbinding sites for transcription factors such as STAT1, STAT10, Sox17, Sox2, and Klf5 (Fig. 2f). These transcription factors are involved in stem cell maintenance and differentiation. In this study, homologous recombination and seamless cloning were performed on five sites to make point mutations, and the promoter activity of the *C2EIP* gene was tested after mutation at each transcription factor binding site. The results showed that increased STAT1 expression can increase the activation of normal PGL3.0/170 (142.54 ± 2.71), while STAT1 binding site mutation can significantly reduce the PGL3.0/170 promoter activity (68.34 ± 2.62) (Fig. 2g).The results for STAT10, Sox17, Sox2, and Klf5 and their binding sites were the opposite (Supplementary Figure 2d-f). Therefore, the transcription factor STAT1 appears to be a positive regulator of the *C2EIP* promoter, whereas STAT10, Sox17, Sox2, and Klf5 are negative regulators. Together, our results indicate that STAT1 binding, histone acetylation, and promoter methylation regulate *C2EIP* expression.

### *C2EIP* promotes PGC generation in vitro and in vivo

**Fig. 3:**
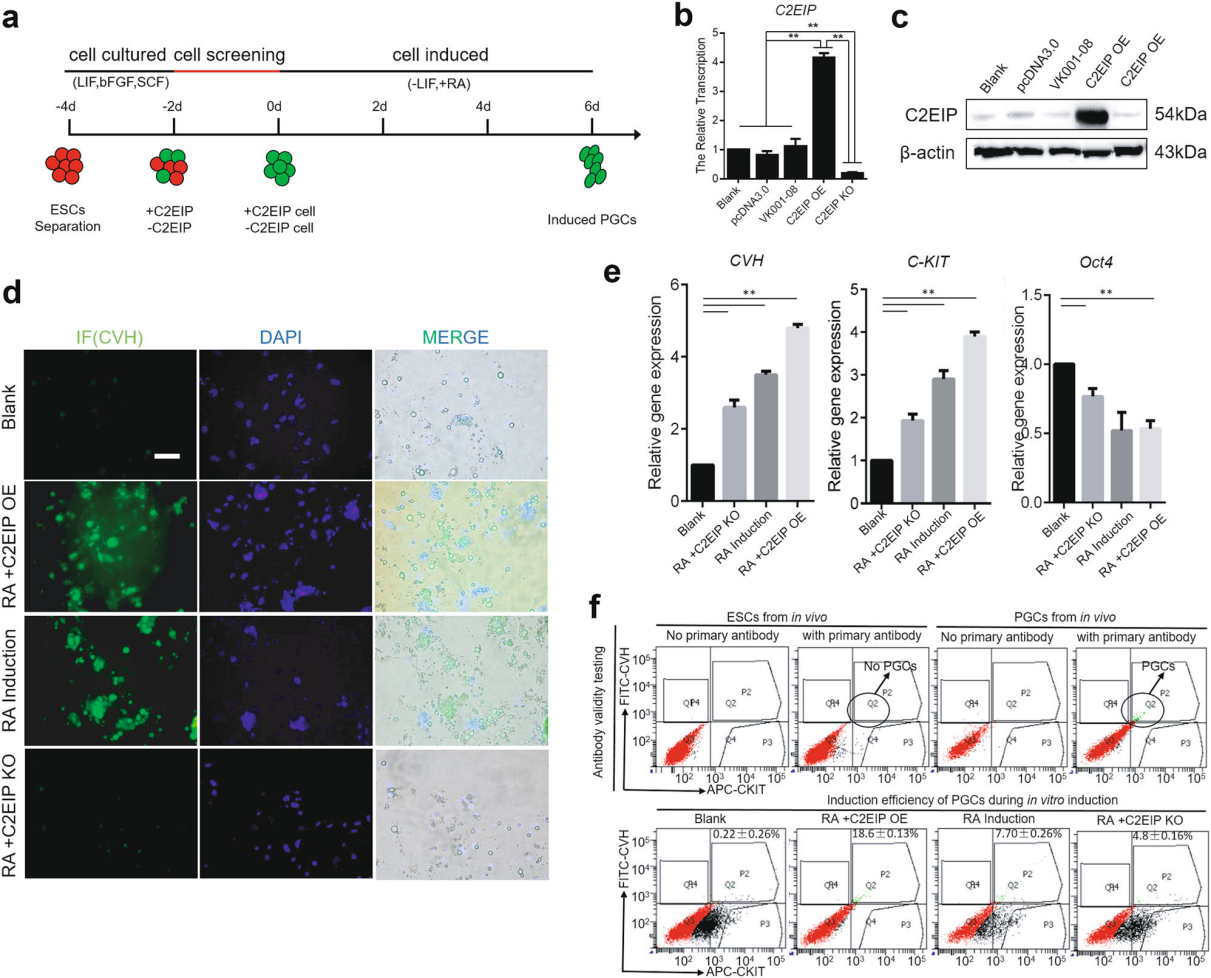
*C2EIP* promotes PGC generation in vitro. **a** Schematic diagram of RA induction model and *C2EIP* modification. Expression of C2EIP mRNA and protein in the different RA induction models was detected by qRT-PCR (**b**) and western blot (**c**), respectively. C2EIP OE and KO groups represent ESC transfected with C2EIP overexpression and knockout vectors, respectively. ESC mock-transfected with no plasmid were the blank control. **d** Fluorescence microscopy after 4–6 days showing PGC-like CVH-positive cells. ESC without RA induction was the negative control, ESC with RA induction and no transfection was the positive control, Scale bar:60μm. **e** qRT-PCR was used to quantify CVH, C-KIT, and OCT4 expression after C2EIP knockout or overexpression. **f** Antibody-specific detection of CVH and C-KIT by flow cytometry. ESC without RA induction was the negative control, and ESC with RA induction and no transfection was the positive controls

To study the role of C2EIP in PGC generation, we implemented a model for inducing ESC differentiation into PGC by in vitro RA treatment (Fig. 3a) (Supplementary Figure 3a-f)^20^, in which we overexpressed *C2EIP* or generated a *C2EIP* knockout via CRISPR/Cas9 (Figs. 3b, c). These experiments showed that *C2EIP* overexpression promoted formation of PGC-like cells (Fig. 3d) (Supplementary Figure 3a, b). Expression levels of *C-KIT* and *CVH*^21,22^, which encode gene products involved in PGC development, were markedly increased in the overexpression group (+2.3-fold; *p* < 0.01), whereas their expression in the knockout group decreased 1.1-fold (*p* < 0.01) compared to controls (Fig. 3e). Flow cytometry analysis showed fewer CVH^+^ cells in the *C2EIP* knockout group (4.8 ± 0.16%; *p* < 0.05) but increased numbers of CVH^+^ cells in the overexpression group (18.6 ± 0.13%; *p* < 0.01) (Fig. 3f). Our results suggest that in vitro* C2EIP* overexpression positively regulates PGC generation.

**Fig. 4:**
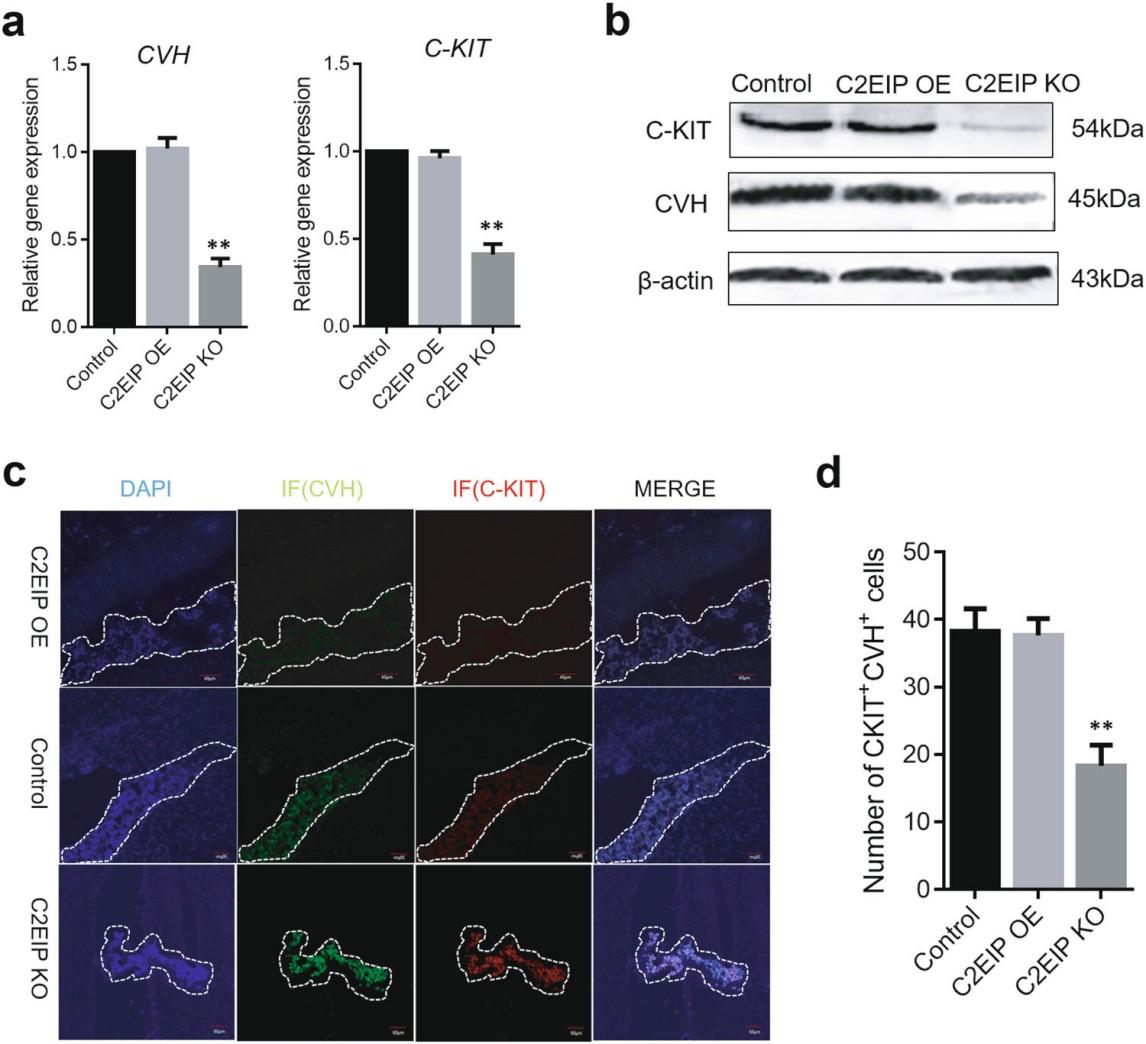
*C2EIP* promotes PGC generation in vivo. **a** Gene expression of *CVH*, and *CKIT* was quantified by qRT-PCR. **b** Protein expression of CVH, and C-KIT was assessed by western blot analysis. Chicken embryos without any treatment were used as controls. β-actin was used as an internal reference. **c** Immunofluorescence of the genital ridge in control and *C2EIP* overexpression (OE) and knockout (KO) hatching chicken embryos. Chicken embryos without any treatment were used as controls, Scale bar: 40 μm. **d** CKIT^+^CVH^+^ cells were quantified using flow cytometry in the indicated groups. Chicken embryos without any treatment were used as controls

To further investigate the function of *C2EIP* during in vivo PGC differentiation, we injected the overexpression orknockout vector, mixed isovolumetrically with polyethylenimine (PEI), into chick embryos (Supplementary Figure 4). The body weight of 4.5-day-old chick embryos in the knockout group was significantly lower than that in the control and overexpression groups^14^, suggesting that *C2EIP* knockout negatively affects chick embryo development. qRT-PCR and Western blot results showed that expression of CVH in the knockout group was reduced compared to the control group (0.31 ± 0.04; *p* < 0.01) (Figs. 4a, b), while CVH expression in the *C2EIP* overexpression group increased compared to the C2EIP KO group (1.03 ± 0.34; *p* < 0.01) (Figs. 4a, b). Moreover, genital glands were observed in control and overexpression group embryos, while no complete genital glands were seen in embryos from the knockout group (Figs. 4c, d).

### Low *C2EIP* expression maintains ESC pluripotency and inhibits PGC generation

**Fig. 5:**
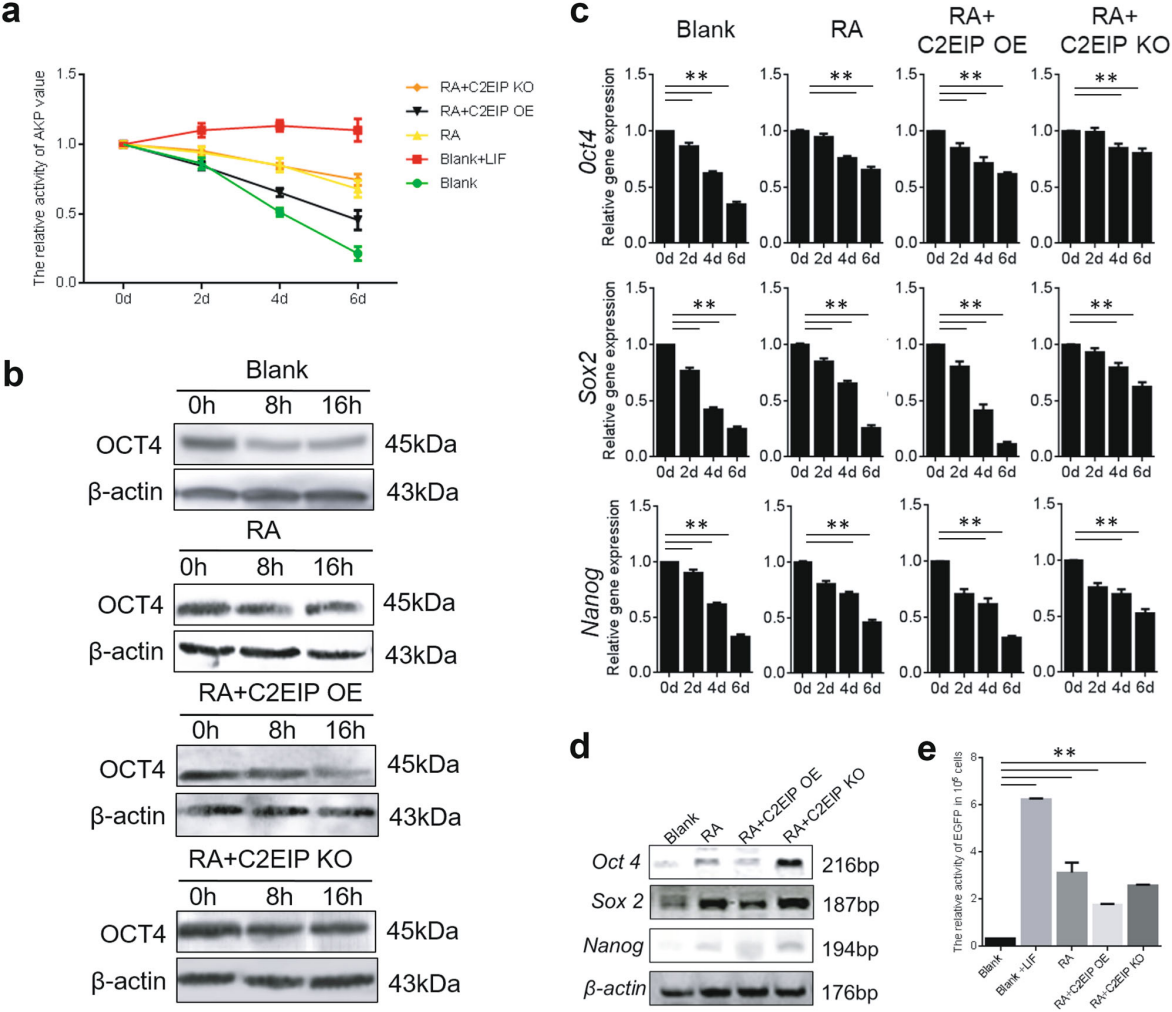
Low *C2EIP* expression maintains ESC pluripotency and inhibits PGC generation. **a** Relative AKP activity was measured in ESC after transfection with *C2EIP* overexpression (OE) or knockout (KO)vector. ESC culture with LIF was the positive control, and ESC culture with no transfection or treatment was the blank control. **b** Western blot analysis of OCT4 protein levels in the RA-induced model. ESC without any treatment were the blank control. **c** qRT-PCR to examine the effects of overexpression and knockout of*C2EIP* on the expression of pluripotency genes. ESC without any treatment were the blank control. **d** mRNA levels of pluripotency genes were detected on day 6 after induction by qRT-PCR. ESC without any treatment were the blank control. **e** RA-induced cells were transfected with N1-pNanog-EGFP vector on day 6 to quantify Nanog promoter-activated EGFP expression. ESC culture with LIF was the positive control, ESC culture with no transfection or treatment was the blank control

We preliminarily analyzed the mechanism underlying *C2EIP*-mediated PGC generation by transfecting ESC with either the *C2EIP* overexpression construct or the CRISPR/Cas9-mediated knockout vector in in vitro RA induction assays. Overexpression of *C2EIP* down-regulated pluripotency-related genes *Oct4* and *Sox2* (Fig. 5a-d)^23,24^, reduced AKP activity (Fig. 5a), and reduced Nanog promoter-activated EGFP expression(1.82 ± 0.06; *p* < 0.01) (Fig. 5e), consistent with the increased PGC generation (Fig. 3e). In contrast, *C2EIP* knockout led to significant increases in *Oct4* and *Sox2* levels, AKP activity, and Nanog promoter-driven EGFP expression (2.41 ± 0.04; *p* < 0.01), respectively (Fig. 5e). With the effects of the overexpression and knockout constructs on expression levels of *Oct4* and *Sox2* in the genital ridge of 4.5-day-old chick embryos(data not shown) were similar to those in the in vitro model experiment. Thus, expression of *C2EIP* appears to limit ESC pluripotency and promote their differentiation into PGC.

### C2EIP and PTCH2 are co-expressed at the intracellular membrane

**Fig. 6:**
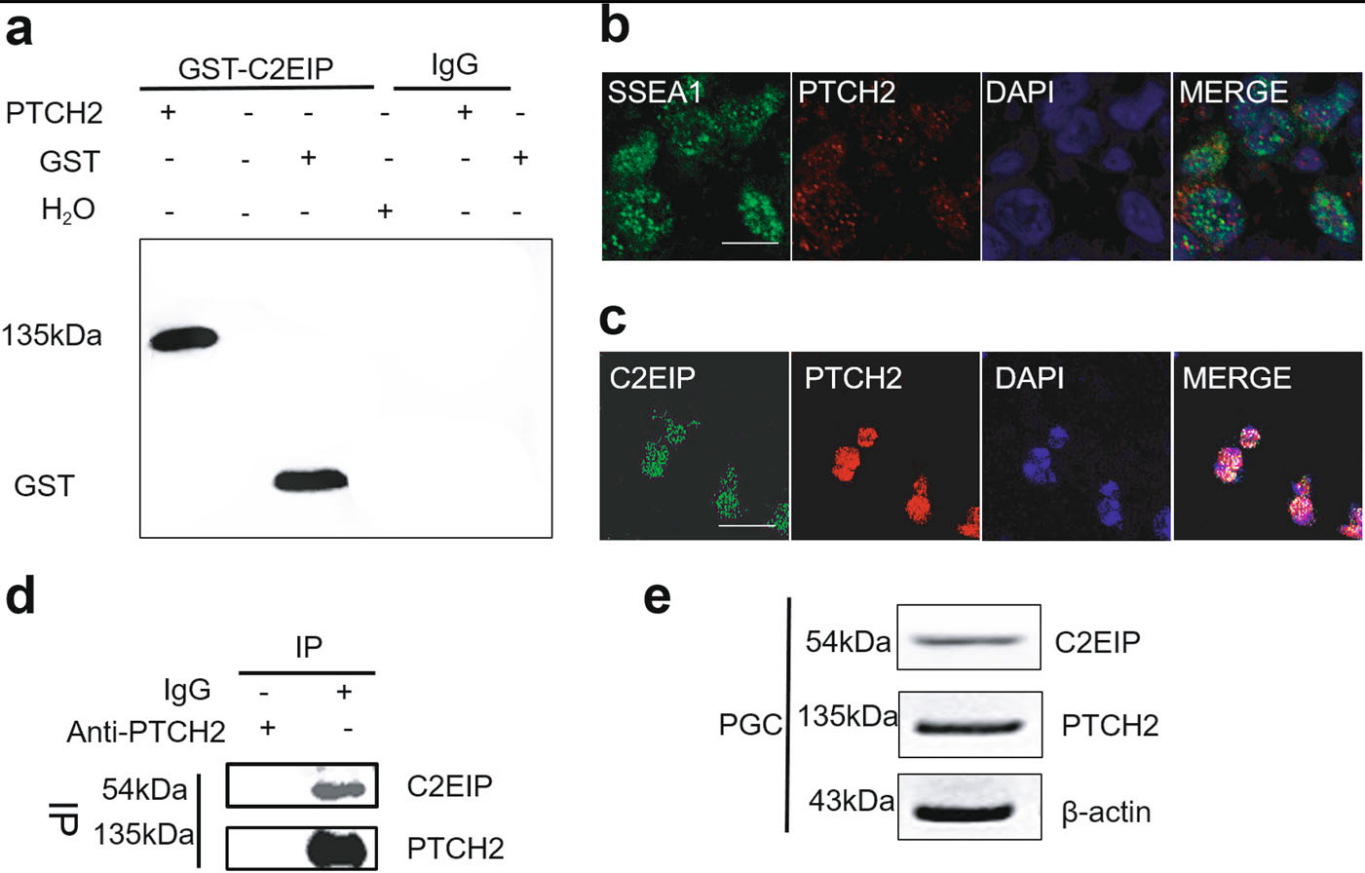
C2EIP and PTCH2 are co-expressed at the intracellular membrane. **a** Interaction between PTCH2 and C2EIP proteins was observed by GSTpull-down test. IgG antibody was used as a negative control. **b** Indirect immunofluorescence results indicate that PTCH2 protein is localized in cell membranes, Scale bar: 30 μm. SSEA-1was used as acell membrane marker. **c** Indirect immunofluorescence results indicate that PTCH2 and C2EIP colocalize at cell membranes, Scale bar: 20 μm. **d** Co-immunoprecipitation from PGC detected an interaction between GST-C2EIP and PTCH2 proteins. IgG antibody was used as negative control. **e** Western blot analysis showed that GST-C2EIP and PTCH2 are expressed in the cytoplasm of PGC

We investigated the molecular mechanism underlying C2EIP-mediated PGC generation by characterizing the C2EIP-PTCH2 interaction with GST pull-down assays and mass spectrum analysis (Supplementary Table 1) (Supplementary Figure 5a-d). Co-immunoprecipitation (co-IP) experiments confirmed the protein-protein interaction (Fig. 6a). Our indirect immunofluorescence assaysfound that PTCH2 colocalized with known membrane protein SSEA-1, indicating that PTCH2 is a membrane protein^25^, whereas C2EIP is primarily localized in the cytoplasm (Figs. 6b, c). C2EIP and PTCH2 form a complex at the intracellular membrane and likely function collectively at that location (Fig. 6c). Western blotting detected C2EIP in the GST-PTCH2 eluate from PGC lysate, suggesting that C2EIP and PTCH2 colocalize in the intracellular membrane of PGC (Figs. 6b-e).

### C2EIP regulates PGC generation by activating the HH signaling pathway

HH signaling is a classical pathway associated with stem cell differentiation that relies on PTCH2 as a membrane surface receptor for regulation^26,27^. When induction molecules are absent, PTCH2 binds to SMO and inhibits HH signaling. In the presence of signaling molecules, C2EIP can bind preferentially, but randomly, to PTCH2, thereby excluding SMO and activating HH signaling. Here, we found that C2EIP interacts with PTCH2 at the intracellular membrane and thus speculated that C2EIP may regulate PGC differentiation by influencing HH signaling pathway activation. Therefore, we constructed lentiviral shRNA-IHH^15^ and shRNA-PTCH2 vectors to investigate the function of HH signaling in the process of PGC generation (Supplementary Figure 6a-c). ESC were transfected with shRNA-IHH or shRNA-PTCH2 in parallel with in vitro RA induction experiments. When ESC were routinely induced by RA, many PGC-like cells emerged 4–6 days after induction; numbers of PGC-like cells did not differ significantly between the IHH and PTCH2 knockdown groups. After IHH knockdown, PGC generation declined, and expression levels of C-KIT and CVH were significantly down-regulated compared to cells in the RA induction group. In contrast, PGC generation and expression of both C-KIT and CVH increased after PTCH2 knockdown (Supplementary Figure 6d-f), which was consistent with the results of our in vivo experiment (Supplementary Figure 7a, b). Thus, PGC generation appears to depend on C2EIP-PTCH2 interactions that lead to activation of the HH signaling pathway.

### C2EIP regulates PGC generation by promoting ubiquitination and dephosphorylation of PTCH2

**Fig. 7:**
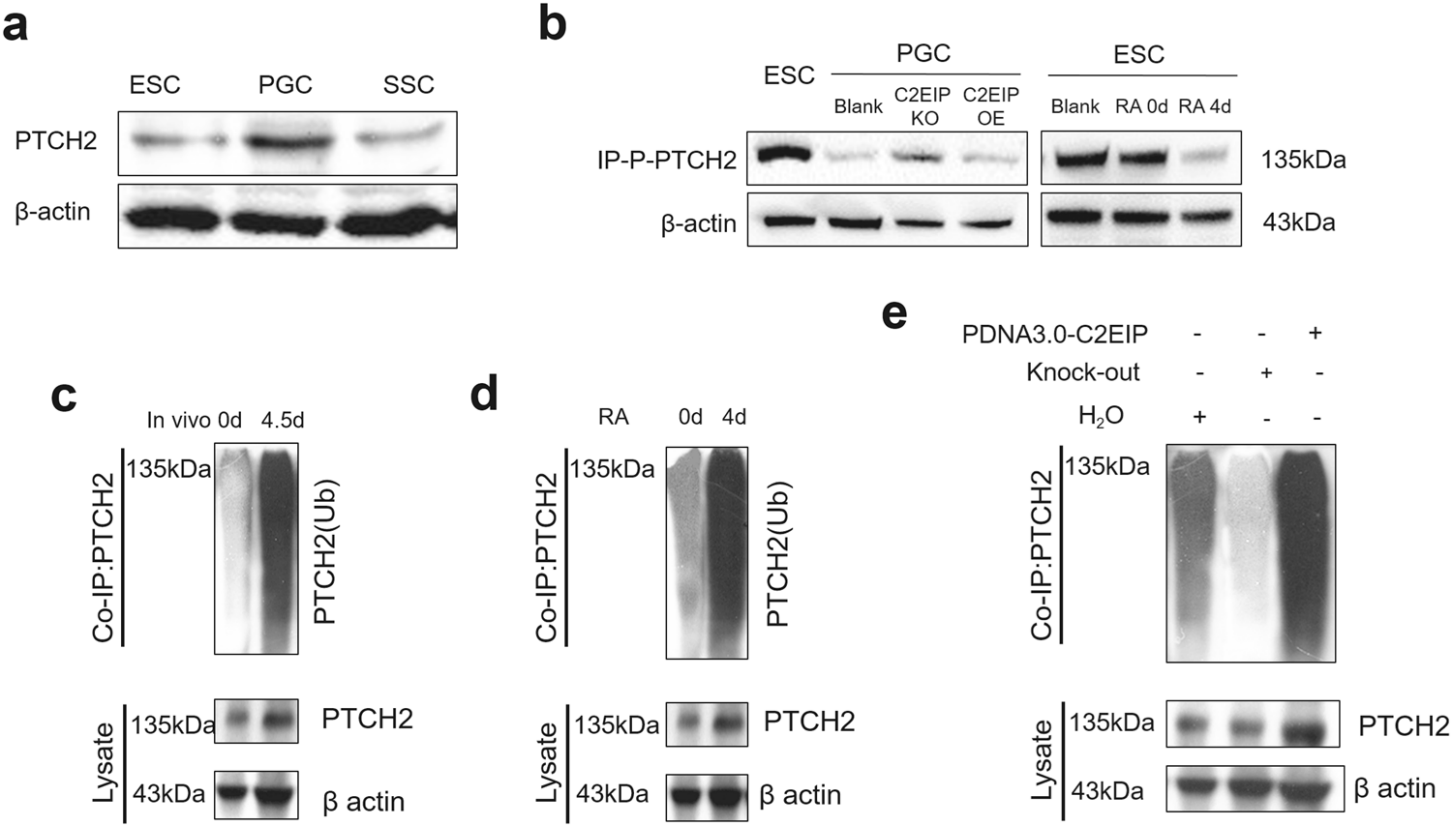
C2EIP regulates PGC generation by activating the HH signaling pathway. **a** Expression of PTCH2 protein in ESC, PGC, and SSC was tested by western Blot. **b** The phosphorylation level of PTCH2 was revealed by co-IP in ESC, PGC, and SSC before and after C2EIP knockout, as well as in the 0-day and 4-day RA induction in vitro model. The phosphorylation level of PTCH2 in ESC was the control. **c**, **d**, **e** The PTCH2 ubiquitylation level was revealed by co-IPin ESC, PGC, and SSC before and after C2EIP knockout as well as in the 0-day and 4-day RA in vitro induction test

We previously confirmed that HH signaling participates in ESC differentiation into PGC. To explore the specific mechanism underlying the relationship between C2EIP-mediated activation of PTCH2 and HH signaling, we measured changes in epigenetic modification of PTCH2 during PGC generation. During the differentiation process, mRNA expression levels of *C2EIP*, *PTCH2*, and *SMO* increased, and the HH signaling pathway was activated, although transcription levels of *C2EIP*, *PTCH2*, and *SMO* were not consistent with their translational levels. Thus, PTCH2 protein might be post translationally modified, leading to reduced activity. Our ubiquitination and dephosphorylation experiments showed decreased dephosphorylation and increased ubiquitination of PTCH2 during ESC differentiation into PGC (Fig. 7a). We observed similar results in the in vitro RA induction experiments (Figs. 7c, d), leading us to conclude that the HH signaling pathway is activated following C2EIP-mediated modification of PTCH2 during PGC generation.

To further define the correlation between C2EIP and the ubiquitination and dephosphorylation of PTCH2, we overexpressed and knocked out C2EIP in PGC and examined the posttranslational modification of PTCH2. When C2EIP was overexpressed, PTCH2 phosphorylation was reduced (data not shown) and ubiquitination increased (Fig. 7e), and phosphorylation of SMO protein was upregulated (data not shown). When C2EIP was knocked out, constitutive phosphorylation of PTCH2 was restored (Fig. 7b) but de-ubiquitination occurred (Fig. 7e), and phosphorylation of SMO decreased(data not shown). Therefore, C2EIP might regulate PGC generation by promoting PTCH2 ubiquitination, excluding PTCH2-SMO interactions, and subsequently activating the HH signaling pathway.

## Discussion

We determined that *C2EIP* is a genetic marker expressed specifically in PGC and can thus complement the use of *CVH* as a differentiation indicator, because *CVH* influences migration, aggregation, and localization of PGC but is not specifically expressed in these cells. *C2EIP* also plays an important role in the late stage of germ cell generation. Like *CVH*, *C-KIT* also contributes to PGC development but is widely expressed in germ cell lines. We utilized high-throughput sequencing and bioinformatics analysis to identify multiple PGC-specific markers, including* Blimp1*, *Prdm14*, and *STELLA*, but focused on the functional role of *C2EIP* in PGC generation and found that its gene product inhibits ESC pluripotency and directs their differentiation into PGC.

The process of ESC-to-PGC differentiation is regulated by both genetic and cell-signaling mechanisms. For example, BMP4^27,28^ and NANOS2^29,30^ can regulate the proliferation and differentiation of PGC by activating the TGF-β/Smad and RA signaling pathways, respectively. The HH signaling pathway, a classical stem cell regulation pathway, regulates cell differentiation by modulating downstream signals through epigenetic modifications. Specifically, IHH binds to PTCH, which leads to SMO release and activation of the HH signal. The PTCH membrane protein is composed of PTCH1 and PTCH2, which share similar ligand binding affinities but often display different expression patterns. PTCH1 functions mainly in somatic cells, while PTCH2 functions mainly in embryo development^14^. If both subunits are highly expressed, HH signaling is inhibited, but lack of expression leads to HH signal activation^31^. During male germ cell differentiation in chick embryos, we observed upregulation of *PTCH1* and downregulation of *PTCH2*, thus highlighting the specific importance of PTCH2 in HH signal activation in ESC. Based on its functional characteristics, we questioned if *C2EIP* is an HH gene family homolog, although subsequent bioinformatic analysis revealed no conserved structural or functional domains between *C2EIP* and the HH gene family and identified different localization patterns. Thus, we began to explore the interaction between PTCH2 and C2EIP and its effect on HH signaling activation. During ESC differentiation, we determined that C2EIP induces the ubiquitination of PTCH2 protein and activates SMO and HH signaling, which together promote PGC generation.

Epigenetic modification is a common mechanism for signaling pathway regulation^32^. For example, ubiquitination and dephosphorylation of Smad proteins modulate TGF-β/BMP signals, while the HH signaling pathway enables phosphorylation of Smad5 and induces signals for downstream cell differentiation^33,34^. We demonstrated that C2EIP specifically activates the HH signal in ESC and promotes their differentiation into PGC. Expression of *C2EIP* and *PTCH2* in ESC is very low initially but increases as numbers of PGC increase, thus perpetuating the HH signal. Our co-IP and in vitro ubiquitination experiments revealed that during the PGC differentiation, phosphorylation of PTCH2 was constantly reduced, while its ubiquitination constantly increased, indicating activation of HH signaling pathway. Thus, these two types of epigenetic modification can reduce accumulated intracellular PTCH2, release SMO protein, transduce the HH signal, promote ESC differentiation, and positively regulate PGC generation.

*C2EIP* is differentially expressed in various cell types, and its promoter plays very important roles in expression modulation. Although *C2EIP* does not include a binding element of RNA polymerase II or a CpG island, the gene is regulated by promoter methylation, histone acetylation, and binding of the STAT1 transcriptional factor in the core area to enhance gene expression. Previous research determined that cell differentiation is negatively affected in STAT1-deficient mice and that cell differentiation of human promyelocytes is related to phosphorylation of STAT1^35^. When we mutated *STAT1*, activity of the *C2EIP* promoter was significantly reduced but could be restored in ESC by RA induction in vitro.

In summary, we identified a PGC-specific marker gene in chick embryos and determined its role in a novel molecular mechanism responsible for directed ESC differentiation. At low methylation levels, STAT1 promotes expression of *C2EIP*, whose gene product binds to PTCH2, induces its ubiquitination, activates the HH signaling pathway, and positively regulates PGC generation.

## Materials and methods

### Cell culture and induction

ESC and PGC were cultured under previously described conditions^36–39^. Well-grown second-generation ESC cells were used in the cell induction experiments^40^. Induction medium was DMEM supplemented with10 mM RA and 15% FBS. To investigate the effects of *C2EIP* gene expression on PGC generation in vitro, ESC were transfected with*C2EIP*overexpression and knockout vectors during induction.

### Antibodies and reagents

Anti-C2EIP mouse antibody (previously used in^14^) was used to detect the expression of C2EIP protein in different tissues. In the flow cytometry and immunohistochemical assays, antibodies against CVH (Abcam, San Francisco, USA; ab27591) and C-KIT (Abcam, San Francisco, USA; ab32363) were used to label PGC. Antibodies against GST (Abcam, San Francisco, USA; ab19256) and C2EIP were used to test the accuracy of the GST pull-down experiment. PTCH2 antibody (Abcam, San Francisco, USA; ab194574) was used to examine protein cellular localization. Antibodies against PTCH2 (Abcam, San Francisco, USA; ab151775) were used for theco-IP experiment. Ubiquitylation antibody (Abcam, San Francisco, USA; ab139467) was used to determinethe ubiquitylation level of PTCH2under different treatments. TSA (MCE, Monmouth, USA; HY-15144) and 5-Zacd (MCE, Monmouth, USA; HY-10586)were used to inhibit deacetylation and methylation, respectively. The dual fluorescein test kit (Solarbio, Beijing, China; D0010) was used to measure the activity of promoter fragments.

### Fluorescence-activated cell sorting

The direct labeling method was used to label the cells. The 4–6 d cells were harvested by transferring suspension to 1.5-mL centrifugal tubes and centrifuging at 1000×*g* for 5 min, then rinsed with PBS 1–2 times and incubated at 4 °C for 1–2 h with 200 μL of 1:200 fluorescein-labeled antibody (CVH or C-KIT). Cells were rinsed with chilled PBS to remove the unbound antibody. Finally, cells were resuspended in 500 μL of chilled PBS. The resultant sample was subjected to flow cytometry analysis. Cells transfected by EGFP fluorescent plasmids were counted and 10^5^ cells were subjected to flow cytometry analysis. For chick embryo cells, the genital ridge was removed after 4.5d incubation (*n* = 30 per group). Single-cell suspension was obtained by trypsin treatment and the cells were counted. Using CVH and C-KIT antibodies, 10^5^ cells were labeled. The cells were then subjected to flow cytometry analysis.

### Construction of overexpression and knockout vectors

Based on the CDS of *C2EIP* in the NCBI database, we designed specific primers to clone the full-length CDS and ligated it into the PCDNA3.0 vector (Supplementary Tables 2, 3). To construct the *C2EIP* gene knockout vector, we selected three specific targets at the APMlocus and ligated them into the VK100081 vector based on the sequence of each exon of *C2EIP* in the UCSC database. The knockout efficiency of three knockout vectors was tested by T7EI, SSA, and TA cloning and sequencing^14^.

### Chicken embryo injection and slice preparation

Plasmid was mixed with PEI at the equal volumes, and 100 µl of the mixture (12 µg) was injected into the chicken embryo via the top end^14^. At 4.5 d after injection, frozen and paraffin-embedded slices were prepared. For frozen slices, the embryo was mixed with cryoprotectant and frozen in liquid nitrogen for 15 s. The frozen embryo was warmed up to −20 °C and then 0.7-µm slices were prepared. The slice was sealed with cover glass and real-time fluorescence was observed under an inverted microscope to observe protein expression. To prepare paraffin-embedded slices, embryos were fixed overnight by paraformaldehyde. After dehydration by alcohol, paraffin slices were prepared. The prepared slice was dewaxed, dehydrated, and used for immunohistochemical assays with CVH and C-KIT antibodies and PAS staining.

### Construction of *C2EIP* promoter deletion fragments

Based on bioinformatics analysis of the *C1EIP* promoter in Rugao yellow chickens, we fixed the downstream primer, changed the upstream primer, and designed five pairs of primers to amplify different deletion fragments. We constructed PGL3 and N1-EGFP vector, cleaved the vectors and PCR products with dual enzymes, recovered the cleaved products, and ligated. After confirming enzymes by enzymatic cleavage and sequencing, these deletion fragments were named PGL3-Basic-P1 to PGL3-Basic-P4, and EGFP-N1-P0 (Supplementary Tables 4, 5).

### Promoter activity assays

DF1 cells were transfected with promoter fragment plasmids using Lipofectamine TM 3000 transfection reagent. Control group cells were transfected by pEGFP-Nl plasmid, and blank control cells were not transfected. Well-grown DF1 cells were grown in 24-well plates at a starting density of 2 × 10^5^ cells. The dual luciferase expression vectors or PGL3-Basic were mixed with PRL-SV40 at a ratio of 1:30 and used to co-transfect the DF1 cells. After 24–48 h of co-transfection, the cells were harvested and the activity of each deletion fragment of the *C2EIP* promoter was tested by the dual luciferase reporter gene detection system. TSA or5-Zacdwas added to the culture solution to test the effects of deacetylation or methylation, respectively, on promoter activity.

### GSTPull-down and mass spectrum (MS) analysis

Specific primers were designed by Primer 5.0 software to clone the full-length CDS of *C2EIP* and introduce *Eco*RI and *Hin*dIII cleavage sites. After cleavage and ligation into the Pet-49(b)-GST plasmid, the experimental plasmids were transformed into the *Escherichia coli* BLexpression strain. Expression was induced by IPTG at 18 °C, and the protein was isolated from cells, purified from the precipitate, concentrated, and used in GST pull-down experiments to identify interactions. To prepare samples for MS analysis, protein was alkylated and enzymatically cleaved after GST pull-down, and the sequence of interacting proteins was identified by LC-MS/MS.

### Indirect immunofluorescence assay

Well-grown PGC after 6d induction were fixed by paraformaldehyde for 30 min, rinsed with PBS three times, permeabilized in 0.5% TritonX-100 for 10 min, and rinsed with PBS three times again. After blocking in PBS containing 10% BSA at room temperature for 30 min, PGC were incubated with C2EIP, PTCH2, C-KIT, or CVH antibodies overnight at 4 °C. These cells were then rinsed with PBST three times and incubated in the dark with secondary antibody at 37 °C for 1 h, rinsed by PBST three times again, and sealed by incubation in glycerol. Protein expression was observed under a fluorescence microscope.

### Western blot analysis

ESC, PGC,or SSC from chicken embryo or 6-d-induced cells were collected and lysed in RIPA buffer. A total of 20 μg of protein in 5 μL loading buffer was boiled for 3–5 min to denature proteins and pipetted into each lane. After separation by 10% SDS-PAGE, the protein was transferred to a nitrocellulose membrane by the semi-dry method. The resulting membrane was blocked by TBST containing 5% fetal bovine serum at room temperature for 1 h. Primary antibodies against Smad2, Smad5, and Notch1 were added and the membrane was incubated overnight at 4 °C. The membrane was then incubated with secondary goat anti-rabbit antibody at 37 °C. The protein was visualized colorimetrically by DAB.

### Co-IP test

The genital ridge of 4.5d chicken embryos was isolated under sterile conditions. The single-cell suspension was produced by trypsin treatment and labeled with the CVH and C-KIT antibodies. High-purity PGC were isolated by flow cytometry. The resulting cells were lysed in RIPA buffer and centrifuged at 13000 g at 4 °C for 15 min. The protein was stored at −70 °C. For co-IP, the extracted protein was incubated overnight with an optimal concentration of anti-PTCH2 antibody. The protein was then centrifuged at 1000×*g* at 4 °C for 10 s.

### Data analysis

The promoter activity test, qRT-PCR, and FACS were replicated three times. The results were analyzed by *t*-test using SPSS19.0 software package. *p* < 0.05 was considered significant, and *p* < 0.01 highly significant. Charts were prepared in GraphPad Prism 6.

### Accession Numbers

The SRA accession numbers for the RNA-seq data reported in this paper are: SRR3720923, SRR3720924, and SRR3720925.

## Electronic supplementary material

Supplementary Table 1

Supplementary Table 2

Supplementary Table 3

Supplementary Table 4

Supplementary Table 5

Supplementary Figure1

Supplementary Figure 2

Supplementary Figure3

Supplementary Figure 4

Supplementary Figure 5

Supplementary Figure 6

Supplementary Figure 7

Supplementary figure legends

Supplementary table legends
