# Supplementary material for: Interaction of the primordial germ cell-specific protein C2EIP with PTCH2 directs differentiation of embryonic stem cells via HH signaling activation
Source: Cell Death Dis. 2018 Apr 27;9(5):497. doi: 10.1038/s41419-018-0557-2 (PMC5923244; doi:10.1038/s41419-018-0557-2)
Supplement: Supplementary file 1 — Supplementary Table 1 [file 41419_2018_557_MOESM1_ESM.docx]

Supplementary Table 1 Mass Spectrometry for C2EIP GST Pull Down protein

| Protein | Polypeptide coverage (%) | Number of peptides |
| --- | --- | --- |
| PTCH2 | 26.60% | 14 |
| KRT75 | 19.20% | 8 |
| H2B-VIII | 27% | 7 |
| KRT12 | 14.90% | 7 |
| Histone H3 | 39.70% | 6 |
| SLC25A6 | 26.20% | 3 |
| LYZ | 27.20% | 2 |
| VDAC2 | 15.60% | 1 |
| Histone H1 | 8.40% | 1 |
| EF-1α | 8.90% | 1 |
| KRT19 | 12.50% | 1 |
