# Supplementary material for: Interaction of the primordial germ cell-specific protein C2EIP with PTCH2 directs differentiation of embryonic stem cells via HH signaling activation
Source: Cell Death Dis. 2018 Apr 27;9(5):497. doi: 10.1038/s41419-018-0557-2 (PMC5923244; doi:10.1038/s41419-018-0557-2)
Supplement: Supplementary file 2 — Supplementary Table 2 [file 41419_2018_557_MOESM2_ESM.docx]

Supplementary Table 2 Primer sequence for *C2EIP* gene cloning

| Primer sequence(5'-3') | Fragment(bp) | Tm(℃) |
| --- | --- | --- |
| F:TGGAGCAAGTCCATGGAGCAT | 216 | 52 |
| R:TTTGAGGAACAGAGA GGCTG |  |  |
