# Supplementary material for: Interaction of the primordial germ cell-specific protein C2EIP with PTCH2 directs differentiation of embryonic stem cells via HH signaling activation
Source: Cell Death Dis. 2018 Apr 27;9(5):497. doi: 10.1038/s41419-018-0557-2 (PMC5923244; doi:10.1038/s41419-018-0557-2)
Supplement: Supplementary file 3 — Supplementary Table 3 [file 41419_2018_557_MOESM3_ESM.docx]

Supplementary Table 3 Primer design for *C2EIP* vector construction

| Primer sequence(5'-3') | Fragment(bp) | Application | Tm℃ |
| --- | --- | --- | --- |
| F: CCCAAGCTTGAGGCTATCAAATGGCAG | 731bp | *C2EIP-N1* Vector Construction | 53 |
| R: CCGGAATTCGTCCCCCAATGAAAATAAAT |  |  |  |
| F: CCCAAGCTTGAGGCTATCAAATGGCAG | 731bp | PCDNA3.0-*C2EIP* Vector Construction | 53 |
| R:CCGGAATTCTCACCCAATGAAAATAAAT |  |  |  |
