# Supplementary material for: Interaction of the primordial germ cell-specific protein C2EIP with PTCH2 directs differentiation of embryonic stem cells via HH signaling activation
Source: Cell Death Dis. 2018 Apr 27;9(5):497. doi: 10.1038/s41419-018-0557-2 (PMC5923244; doi:10.1038/s41419-018-0557-2)
Supplement: Supplementary file 4 — Supplementary Table 4 [file 41419_2018_557_MOESM4_ESM.docx]

Supplementary Table 4 Primer design and its application.

| Primer sequence(5'-3') | Restriction sites | Site | Application |
| --- | --- | --- | --- |
| 5' CGCATTAATGTTGATTGTGGGAACTGT 3' | AseI | -1205 | N1-EGFP |
| 5' GGGGTACCAAAGGTTGGAGGAGGTGA 3' | KpnI | -891 | PGL3-Basic |
| 5' GGGGTACCCCATAGTTTGGATGTCTG 3' | KpnI | -682 | PGL3-Basic |
| 5' GGGGTACCACAGAAGAGGGCAAGAGG 3' | KpnI | -486 | PGL3-Basic |
| 5' GGGGTACCATTGGTAGGCAGTGGTGG 3' | KpnI | -264 | PGL3-Basic |
| 5' CCCAAGCTTCTCCACTCAAAGGGTCAG 3' | HindIII | -94 | N1-EGFP and PGL3-Basic |
