# Supplementary material for: Interaction of the primordial germ cell-specific protein C2EIP with PTCH2 directs differentiation of embryonic stem cells via HH signaling activation
Source: Cell Death Dis. 2018 Apr 27;9(5):497. doi: 10.1038/s41419-018-0557-2 (PMC5923244; doi:10.1038/s41419-018-0557-2)
Supplement: Supplementary file 5 — Supplementary Table 5 [file 41419_2018_557_MOESM5_ESM.docx]

Supplementary Table 5 Design of transcription factor point mutation primer

| TF | Primer | Primer sequence(5'-3') | Mutation site |
| --- | --- | --- | --- |
| *STAT1* | F-Kpn I | GAACATTTCTCTATCGATA**GGTACC**CCATAGTTTGGATGTCTG | ATTCCCTGCAT mutation to ATTCGCTGCAT |
|  | *STAT1*-RP | **C**GAATGGGTCTTTTCCACCACTGCCTAC |  |
|  | S*TAT1*-FP | GTGGAAAAGACCCATTC**G**CTGCATTAGAAG |  |
|  | R-HindIII | CAGTACCGGAATGCC**AAGCTT**CTCCACTCAAAGGGTCAG |  |
| *STAT10* | F-Kpn I | GAACATTTCTCTATCGATA**GGTACC**CCATAGTTTGGATGTCTG | TAATTTCACTTAAA mutation to TAATTTCGCTTAAA |
|  | *STAT10*-R1 | CATTTTAAGCGAAATTACTTCTAATG |  |
|  | *STAT10*-F1 | GAAGTAATTTCGCTTAAAATGTATC |  |
|  | R-HindIII | CAGTACCGGAATGCC**AAGCTT**CTCCACTCAAAGGGTCAG |  |
| *SOX17* | F-Kpn I | GAACATTTCTCTATCGATA**GGTACC**CCATAGTTTGGATGTCTG | TTTATTATC mutation to TTTGTTATC |
|  | *SOX17*-R1 | CATCGCACGATAACAAAATAG |  |
|  | *SOX17*-F1 | GAAGTAATTTCGCTTAAAATGTATC |  |
|  | R-HindIII | CAGTACCGGAATGCC**AAGCTT**CTCCACTCAAAGGGTCAG |  |
| *klf* | F-Kpn I | GAACATTTCTCTATCGATA**GGTACC**CCATAGTTTGGATGTCTG | GTGGT mutation to GTAGT |
|  | *klf*-R1 | GTCTTTTCCACTACTGCCTACC |  |
|  | *klf*-F1 | GGTAGGCAGTAGTGGAAAAGAC |  |
|  | R-HindIII | CAGTACCGGAATGCC**AAGCTT**CTCCACTCAAAGGGTCAG |  |
| *SOX2* | F-Kpn I | GAACATTTCTCTATCGATA**GGTACC**CCATAGTTTGGATGTCTG | CCTTTG mutation to CCTGTG |
|  | R-HindIII | CAGTACCGGAATGCC**AAGCTT**CTCCACTCACAGGGTCAGTGCAGCAATAAATG |  |
