# Supplementary figures and images for: Interaction of the primordial germ cell-specific protein C2EIP with PTCH2 directs differentiation of embryonic stem cells via HH signaling activation
Source: Cell Death Dis. 2018 Apr 27;9(5):497. doi: 10.1038/s41419-018-0557-2 (PMC5923244; doi:10.1038/s41419-018-0557-2)

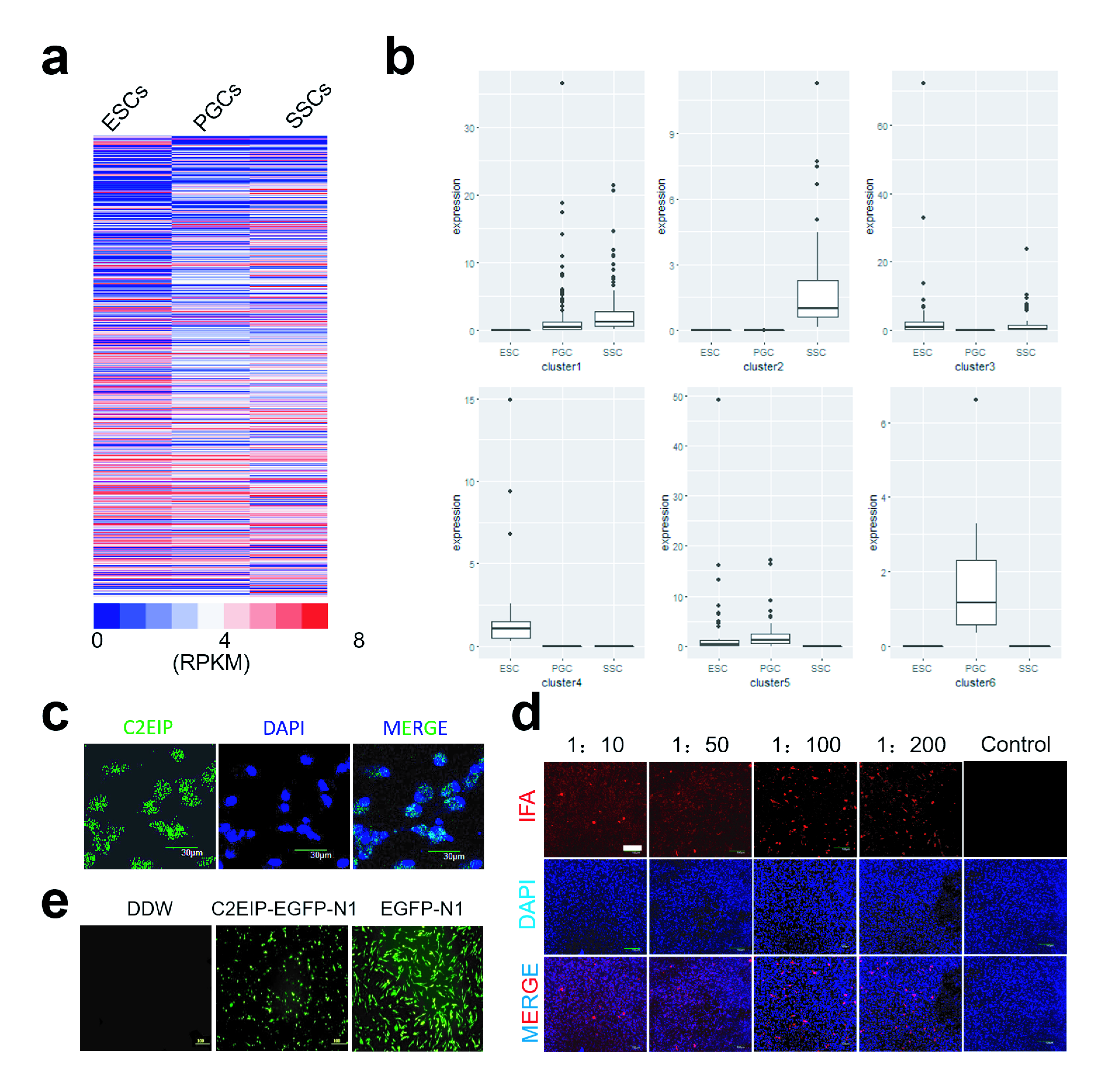

Supplement: Supplementary file 6 — Supplementary Figure1 [file 41419_2018_557_MOESM6_ESM.tif]

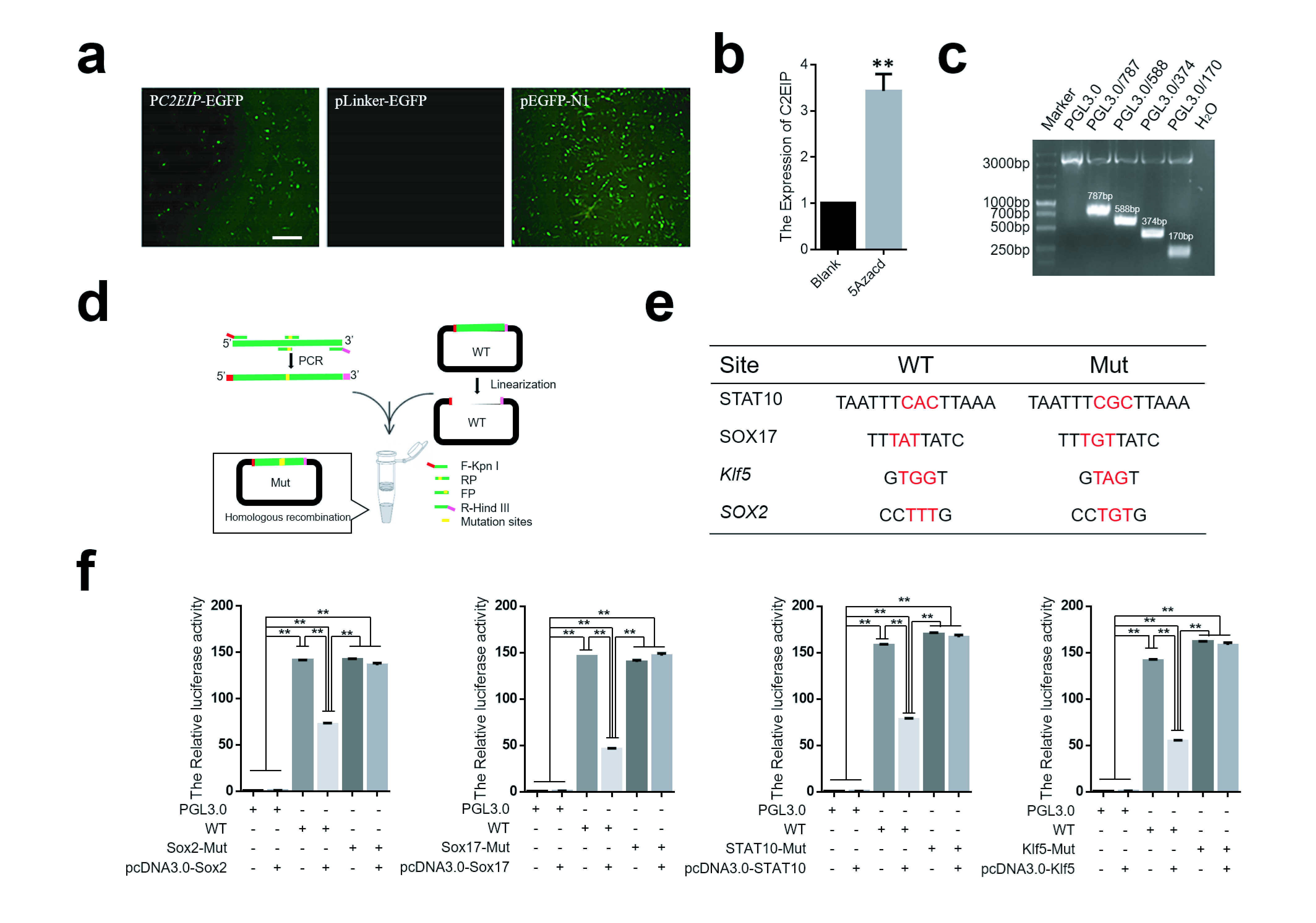

Supplement: Supplementary file 7 — Supplementary Figure 2 [file 41419_2018_557_MOESM7_ESM.tif]

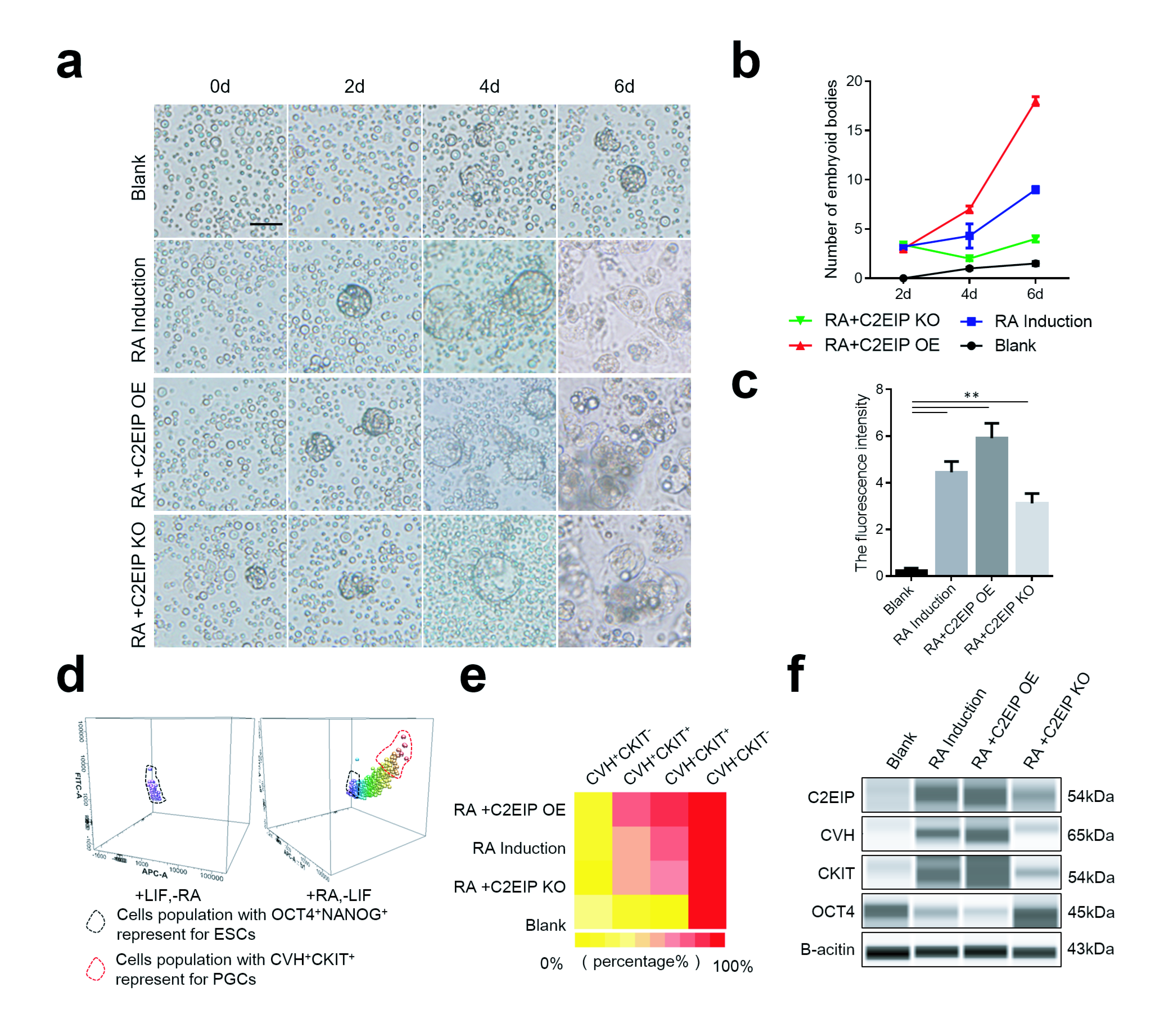

Supplement: Supplementary file 8 — Supplementary Figure3 [file 41419_2018_557_MOESM8_ESM.tif]

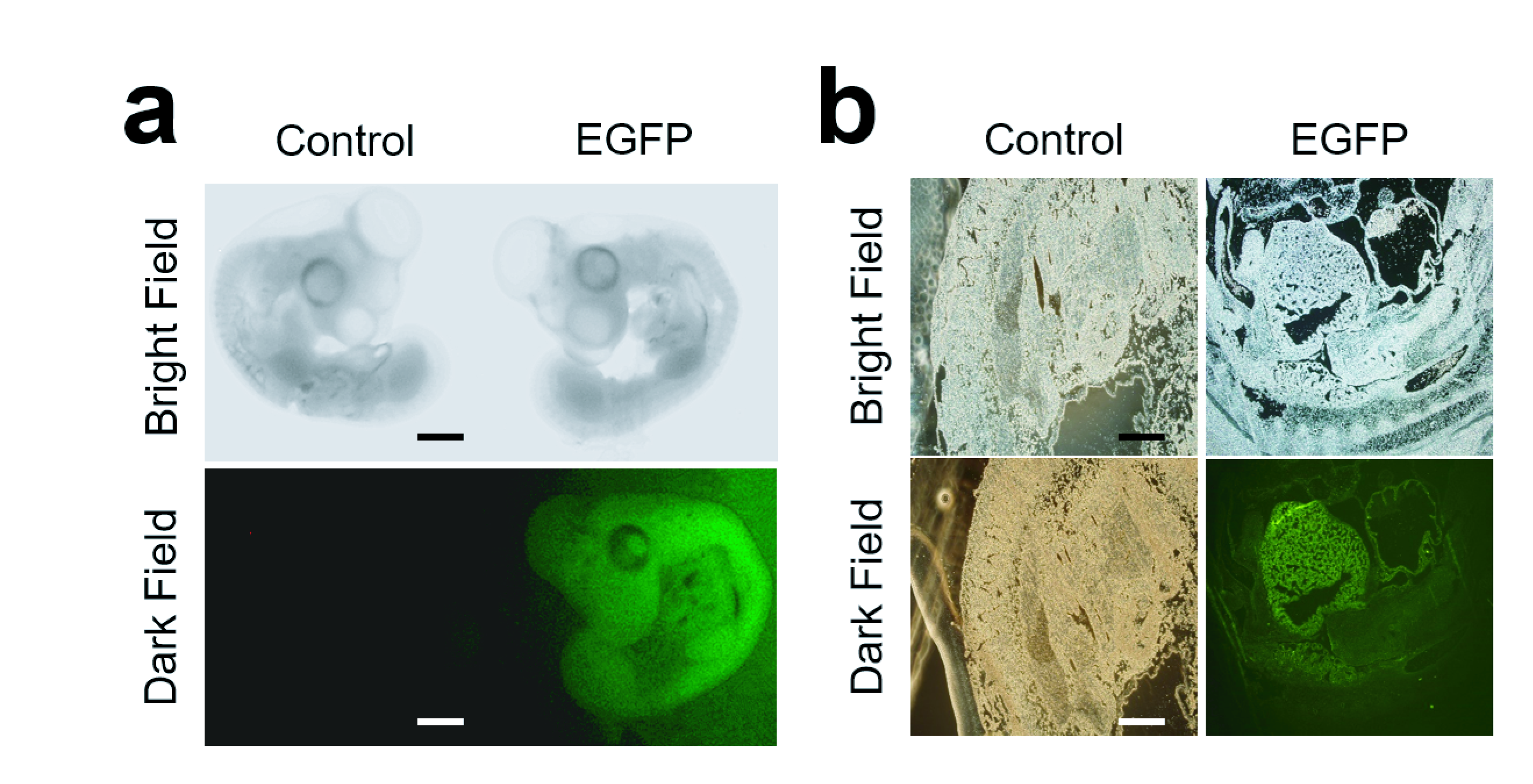

Supplement: Supplementary file 9 — Supplementary Figure 4 [file 41419_2018_557_MOESM9_ESM.tif]

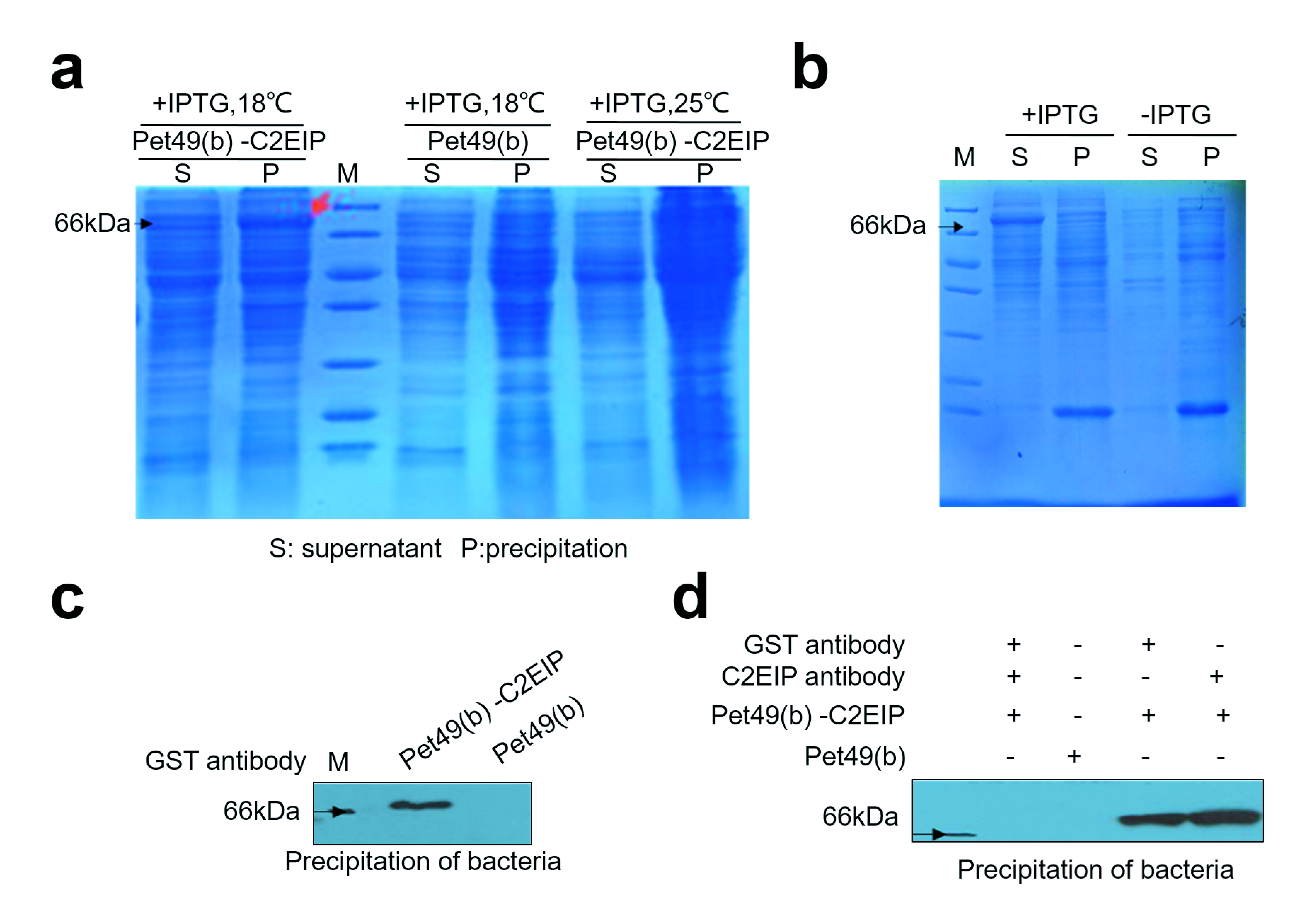

Supplement: Supplementary file 10 — Supplementary Figure 5 [file 41419_2018_557_MOESM10_ESM.tif]

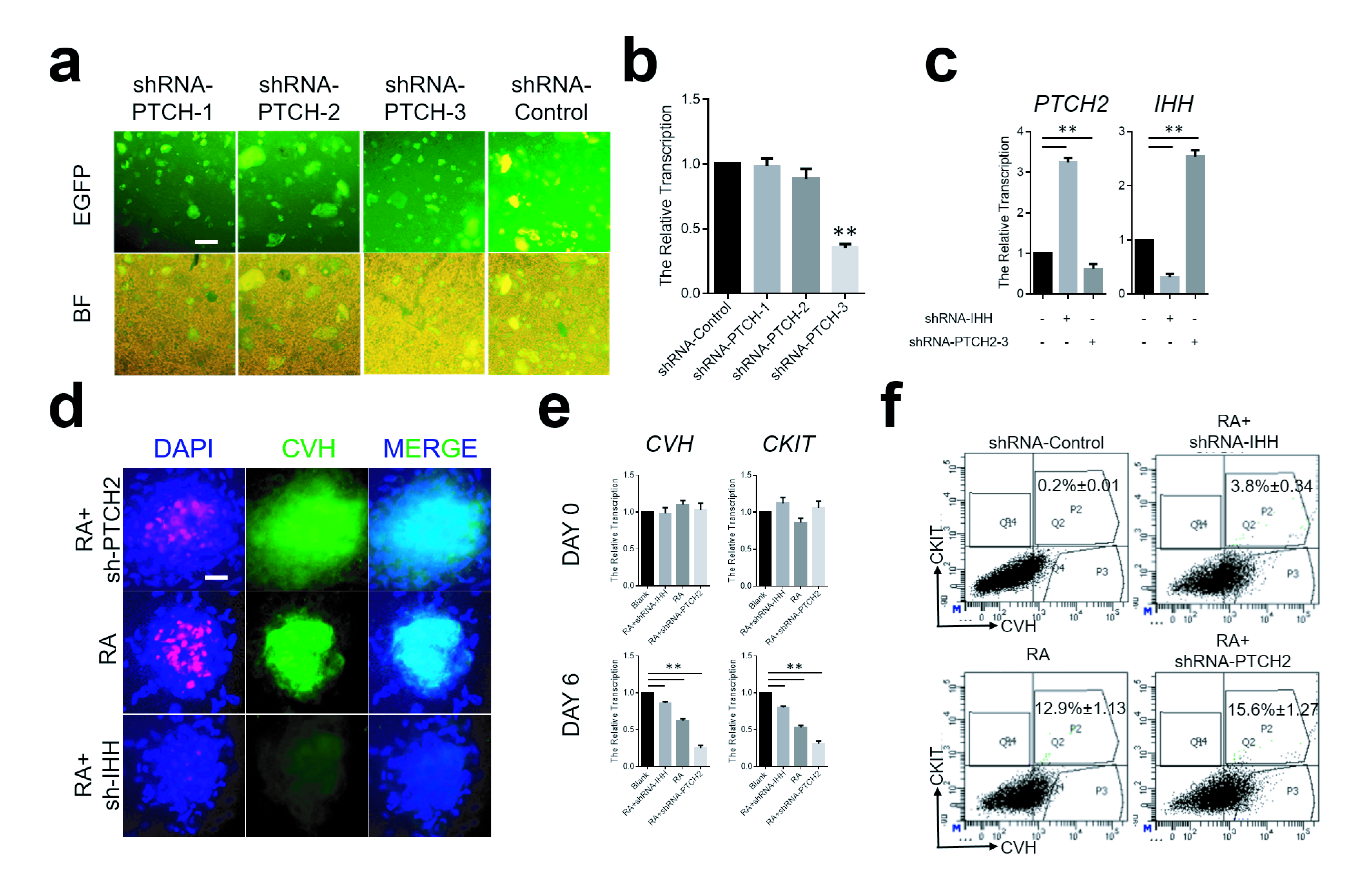

Supplement: Supplementary file 11 — Supplementary Figure 6 [file 41419_2018_557_MOESM11_ESM.tif]

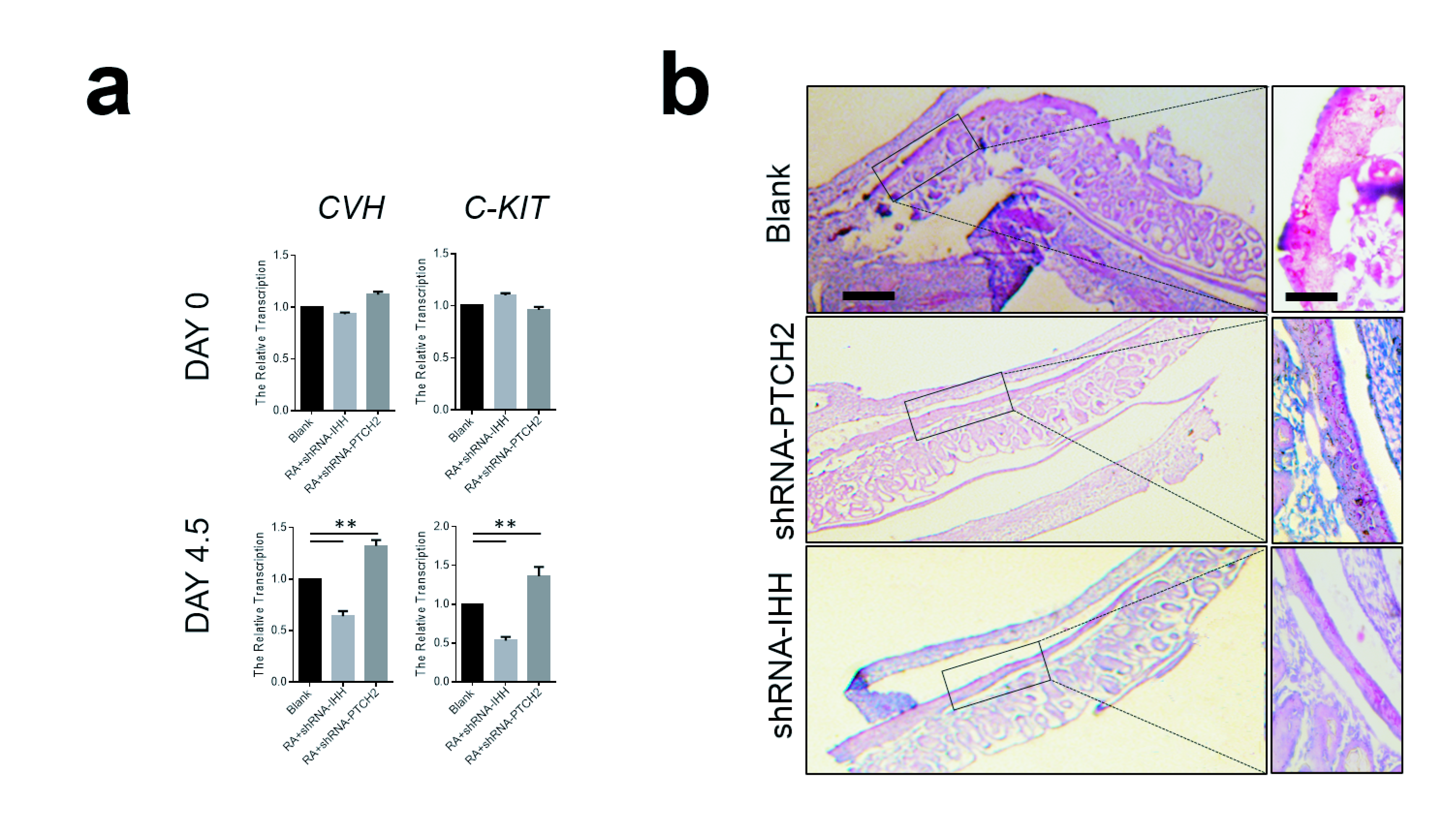

Supplement: Supplementary file 12 — Supplementary Figure 7 [file 41419_2018_557_MOESM12_ESM.tif]
