# Supplementary material for: Interaction of the primordial germ cell-specific protein C2EIP with PTCH2 directs differentiation of embryonic stem cells via HH signaling activation
Source: Cell Death Dis. 2018 Apr 27;9(5):497. doi: 10.1038/s41419-018-0557-2 (PMC5923244; doi:10.1038/s41419-018-0557-2)
Supplement: Supplementary file 13 — Supplementary figure legends [file 41419_2018_557_MOESM13_ESM.docx]

**Supplementary Figure Legends**

**Supplementary Figure1: Screening of *C2EIP*gene expression.**

**a** Clustering analysis of RNA-seq results. **b** Box plot showed that differentially expressed genes can be divided into six clusters (shown in detail in Fig. 1). **c** Indirect immunofluorescence assay showing C2EIP localization in PGC, Scale bar:30μm.**d** IFA results show that the polyclonal antibody titer is 1:10. Cells without antibody were used as a blank control, Scale bar:100μm.**e** C2EIP-EGFP-N1 vector transfection resulted in EGFP expression, Scale bar:100μm. Cells transfected with EGFP-N1 vector were used as a positive control, andcellsmock-transfected with double distilled water (DDW) were used as a negative control, Scale bar:100μm.

**Supplementary Figure 2: Result of transcription factor site mutation.**

**a** EGFP expressionin DF-1 cells after transfection with PC2EIP-EGFP, indicating that the cloned *C2EIP* promoter is active. DF-1 cells transfected with pLinker-EGFP andpEGFP-N1 wereused as negative and positive controls, respectively. Scale bar:100μm.**b** qRT-PCT analysis ofC2EIP expression after addition of 5-Zacd to PGC culture. Untreated cells were used as blank controls. **c** Agarose gel electrophoresis verifying that different length fragments of the *C2EIP* promoterwere cloned successfully. **d,e**Transcription factor binding sites forTAT10, Sox17, Sox2, and Klf5 were mutated. **f** Effect of point mutations of STAT10, Sox17, Sox2, and Klf5 transcription factors on *C2EIP*promoter activity.

**Supplementary Figure3: *C2EIP* promotes PGC generation *in vitro*.**

**a**Morphological changes in RA-induced ESC cells with C2EIP overexpression (OE) or knockout (KO). ESC without RA induction was the blank control, Scale bar:100μm.**b** Number of embryoid bodies formedin cells treated as in **a**. ESC without RA induction was the blank control.**c**ImageJ software was used to calculate the fluorescence intensity of cells treated as in **a**. **d,e**The RA induction model leads to PGC-like cell formation, and this population groups significantly.**f** Western blot analysis of PGC markers in cells treated as in **a**. ESC without RA induction was the blank control.

**Supplementary Figure 4: Exogenous reporter vectors are integrated and expressed in chicken embryos.**

**a S**tereotactic fluorescence microscopy was used to observe the stable expression of exogenous EGFP-expressing reporter vectorduring chicken embryo development.Chicken embryos that had not been injected with an exogenous vector were used as controls, Scale bar:5mm. **b** Frozen sections of 4.5-day-old chicken embryosshowingEGFP expression froman exogenous reporter vector, Scale bar:1mm.

**Supplementary Figure 5:Construction and expression of *C2EIP*prokaryotic fusion expression vector.**

**a** C2EIP protein expression from the Pet49(b)-C2EIP vector was induced by IPTG in *E. coli*BL bacteria (arrows indicate 66-kDa GST-C2EIP).Pet49(b) vector in *E. coli*BL bacteria was the blank control. **b**GST-C2EIP expressed in *E. coli*BL bacteria. **c**Anti-GST antibody was used in western blot analysis to detect the expression of GST-C2EIP. **d**Antibodies against GST and C2EIPwere used to detect the expression of GST-C2EIP.

**Supplementary Figure 6 C2EIP Regulates PGC Generation by Activating the HH Signaling Pathway *in vitro*.**

**a** Expression of EGFP in ESCs transfected with PTCH2 interference expression vector. **b** The expression of PTCH2 in ESCs was detected by qRT-PCR, the results showed that the expression of PTCH2 was significantly inhibited by shRNA-PTCH2-3, Scale bar:30μm.**c** qRT-PCR results showed that shRNA-IHH and shRNA-PTCH2-3 were able to express IHH and PTCH2 genes respectively. **d** On day 6 of the RA induction model: PGC-positive cells produced after transfection with shRNA-IHH were significantly less than those transfected with shRNA-PTCH2-3. Cell induced by RA was regarded as positive control, Scale bar:20μm. **e** qRT-PCR detected the expression of CVH and C-KIT genes on the sixth day of each induction group. The results showed that inhibition of PTCH2 could promote the expression of CVH and C-KIT, while the inhibition of IHH showed the opposite result. **f** Flow cytometric analysis showed that inhibition of PTCH2 expression promoted the formation of PGC, while inhibition of IHH inhibited the formation of PGC.

**Supplementary Figure 7 HH Signaling regulates the formation of PGC *in vivo*.**

**a** During embryo hatching, shRNA-IHH and shRNA-PTCH2 were injected into chicken embryos and hatched normally. After 4.5 days of incubation, PGC from each group were collected. qRT-PCR results showed that inhibition of PTCH2 expression could promote the expression of CVH and C-KIT, while inhibition of IHH expression had the opposite result. **b** 4.5 days after hatching, collecting genital ridges of each group. The results of paraffin section and PAS staining showed that after PTCH2 expression was suppressed, the number of PGC in the genital ridge was significantly more than that of IHH.That is, inhibition of PTCH2 can promote the formation of PGC, and inhibition of IHH can inhibit the formation of PGC.Chicken embryos that have not been treated as blank control. Left Scale bar:200μm , Right Scale bar:40μm.
