# Supplementary material for: Interaction of the primordial germ cell-specific protein C2EIP with PTCH2 directs differentiation of embryonic stem cells via HH signaling activation
Source: Cell Death Dis. 2018 Apr 27;9(5):497. doi: 10.1038/s41419-018-0557-2 (PMC5923244; doi:10.1038/s41419-018-0557-2)
Supplement: Supplementary file 14 — Supplementary table legends [file 41419_2018_557_MOESM14_ESM.docx]

**Supplementary table Legends**

**Supplementary Table 1 Mass Spectrometry forC2EIP GST Pull Down protein**

**Supplementary Table 2 Primer sequence for C2EIP gene cloning**

**Supplementary Table 3 Primer design for C2EIP vector construction**

**Supplementary Table 4 Primer design and its application.**

**Supplementary Table 5 Design of transcription factor point mutation primer**
